# Dental Erosion and Its Growing Importance in Clinical Practice: From Past to Present

**DOI:** 10.1155/2012/632907

**Published:** 2012-03-07

**Authors:** Ann-Katrin Johansson, Ridwaan Omar, Gunnar E. Carlsson, Anders Johansson

**Affiliations:** ^1^Department of Clinical Dentistry—Cariology, Faculty of Medicine and Dentistry, University of Bergen, 5009 Bergen, Norway; ^2^Department of Restorative Sciences, Faculty of Dentistry, Kuwait University, Safat 13110, Kuwait; ^3^Department of Prosthetic Dentistry, The Sahlgrenska Academy at University of Gothenburg, 405 30 Göteborg, Sweden; ^4^Department of Clinical Dentistry—Prosthodontics, Faculty of Medicine and Dentistry, University of Bergen, 5009 Bergen, Norway

## Abstract

Since the mid-1990s, the focus of studies on tooth wear has steadily shifted from the general condition towards the more specific area of dental erosion; equally, a shift has occurred from studies in adults to those in children and adolescents. During this time, understanding of the condition has increased greatly. This paper attempts to provide a critical overview of the development of this body of knowledge, from earlier perceptions to the present. It is accepted that dental erosion has a multifactorial background, in which individual and lifestyle factors have great significance. Notwithstanding methodological differences across studies, data from many countries confirm that dental erosion is common in children and young people, and that, when present, it progresses rapidly. That the condition, and its ramifications, warrants serious consideration in clinical dentistry, is clear. It is important for the oral healthcare team to be able to recognize its early signs and symptoms and to understand its pathogenesis. Preventive strategies are essential ingredients in the management of patients with dental erosion. When necessary, treatment aimed at correcting or improving its effects might best be of a minimally invasive nature. Still, there remains a need for further research to forge better understanding of the subject.

## 1. Introduction

**Figure 1:**
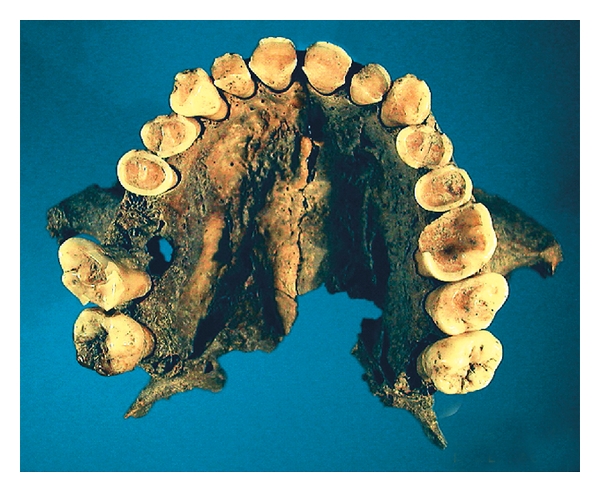
Extensive tooth wear of maxillary teeth in a medieval man estimated to be 35 to 45 years old. The loss of the first right molar was most likely caused by wear penetrating into the pulp subsequently leading to an inflammatory process in the periapical jawbone [6].

Interest in dental erosion and its role in tooth wear increased considerably since the mid-1990s. Early studies on tooth wear in humans were, in the main, based on teeth from archeologically obtained skulls. In later studies, contemporary adult populations were examined, but in neither the early nor the later periods of study, erosion was rarely, if ever, mentioned as a possible etiological factor [1] (Figure 1). The definition and diagnosis of dental erosion have not been agreed upon among researchers and clinicians, which can explain some of the confusion and perhaps the earlier lack of interest in the subject [2]. The diet of our ancestors was often tough and contained coarse particles, which required heavy chewing. The resulting wear facets were further influenced by the abrasive components of the food. Modern diets would appear to lack such abrasives but can contain acids, which can demineralize enamel and potentiate attrition and abrasion. Nevertheless, there are findings supporting the existence of dental erosion even in medieval populations [3, 4].

Although the terms attrition, erosion, and abrasion are the commonly accepted nomenclature used in dentistry to characterize tooth wear, the terms do not explain the wear process. Neither do they imply causation, instead describing clinical outcomes of a number of underlying events. In this regard, the science of tribology may more accurately characterize the process of tooth wear. There are several tribological mechanisms, although the mechanisms that apply in tooth wear may be explained in terms of two-body abrasion (typically attrition) or three-body abrasion interacting with an acidic or abrasive fluid/slurry (typically erosion and abrasion) [5].

**Figure 2:**
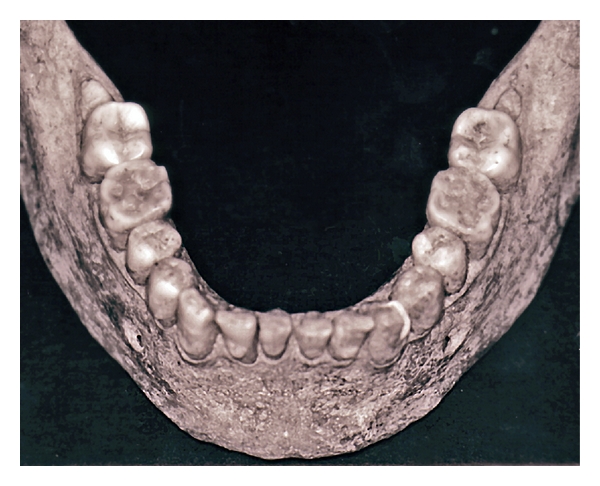
Severe tooth wear on the mandibular first molars in an approximately 20-year-old individual from the 16th century (third molars are impacted). Looking carefully, NCCLs can be seen indicating either abrasive or erosive influences, which, in combination with the wear seen on the first molars, resembles the pattern seen in modern erosive wear [11].

The earliest form of tooth wear was found mainly on occlusal, incisal and proximal surfaces, whereas modern erosive tooth wear has additional characteristics that include the buccal and palatal/lingual surfaces. Tooth wear in archeological material was, from an anthropological point of view, considered pathologic only if the function of the tooth was lost [7]. This may be one reason why dental erosion may have been overlooked as a possible cause in studies of tooth wear in those populations even though its morphological features sometimes have similarities with what we see today (Figure 2).

A further change in emphasis regarding the subject occurred in the mid-1990s: studies on tooth wear shifted from that of adult wear to wear in children and adolescents, as well as from “general tooth wear,” that is, attrition and abrasion, to giving greater attention to the significance of etiological factors resulting in erosive tooth wear or dental erosion. Recent studies from a large number of countries all over the world have confirmed that the prevalence of erosive wear, especially among children and adolescents, is high (Table 1). Besides this, some longitudinal studies show that the occurrence of erosion is increasing and that erosive lesions that are already present progress rapidly [8–10]. Even if the prevalence rates vary substantially, it is evident that dental erosion is a common finding in populations from all over the world, especially among children and adolescents (Table 1). During the past few years, definitions of diagnosis and grading of the erosive lesions have been highlighted, and it is to be hoped that such moves towards greater standardization may lead to greater concordance between studies conducted and the methods used. As a result of these findings, dental erosion deserves serious consideration in clinical dentistry today. Despite the common finding of an increase of tooth wear, especially in the younger population during the last decade, the number of publications in PubMed dealing with, for example, dental caries, surpasses studies on dental erosion by a factor of over 20: over the past 10 years, obtaining 335 hits for dental erosion; 8750 for dental caries.

This paper is not a systematic review, which would have needed a more focused aim than is presently the case. It was also obvious from the literature review conducted that very few papers would have fulfilled the requirements of highest-ranking evidence, according to the hierarchy of evidence-based medicine, that is, randomized controlled trials (RCTs) and/or systematic reviews of RCTs. Given that, many of the selected articles present valuable findings that add to the body of knowledge on dental erosion. It is, therefore, the purpose of this paper to give an overview of current knowledge of dental erosion, based on a scrutiny of the literature.

## 2. Etiology of Dental Erosion

**Figure 3:**
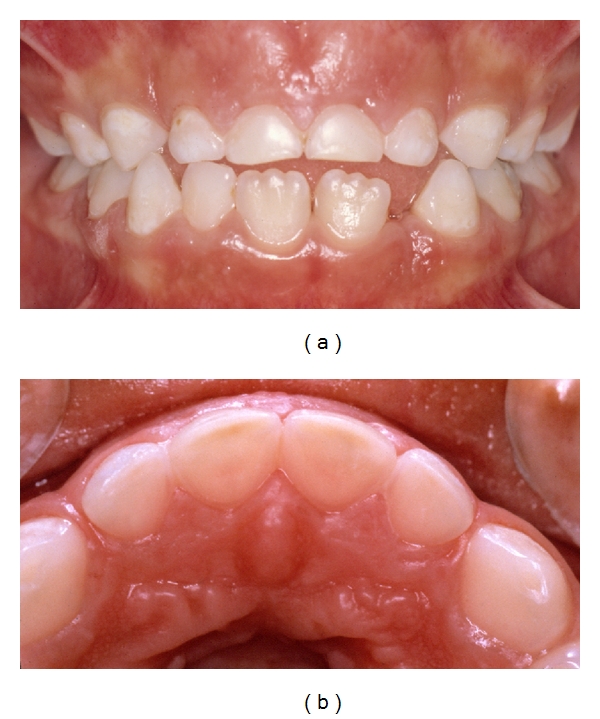
(a) A 6-year-old boy with dental erosion associated with a high intake of soft drinks and juice. Note the “melted” appearance of the buccal surfaces on teeth nos. 51–61. (b) Palatally on the maxillary front teeth the pulp is visible through the remaining tooth substance. Published with permission from the Swedish Dental Journal [2].

**Figure 4:**
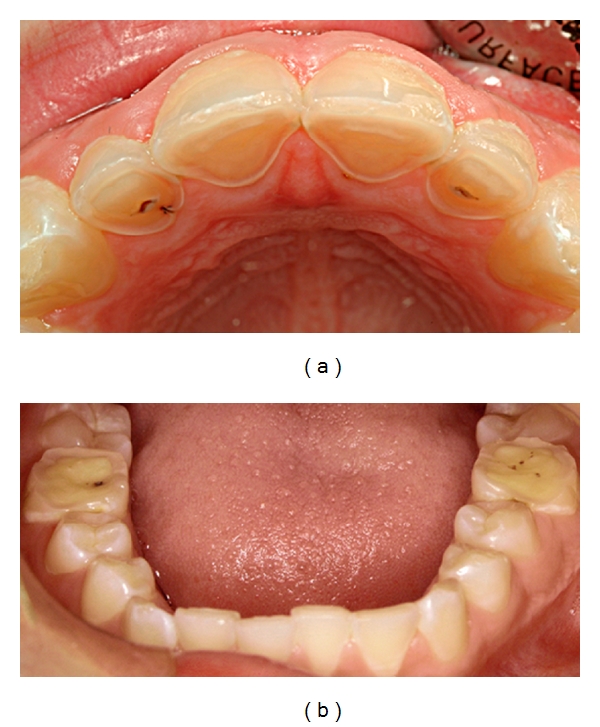
A 12-year-old boy who has a high intake of cola. (a) Note the severe damage with shoulder formations palatally on maxillary front teeth. (b) First molars also exhibit pronounced wear. Published with permission from the Journal of the Swedish Dental Association (Tandläkartidningen) [12].

The etiology of dental erosion is conventionally divided into “extrinsic” and “intrinsic” factors [34]. Any of the acidic products that we put into the mouth, that is, what we eat and drink, and also what has been termed occupational-related erosion, often caused by airborne acid that reaches the teeth, for example, in workers in certain industries, or people who are wine taster [35–40], are considered “extrinsic” factors. There has been a considerable increase in the intake of soft drinks in recent decades, and these often are high in acidic content [41]. It is clear that, in children and adolescents today, the dominant causative factor for erosion is soft drinks [25, 42–44] (Figures 3 and 4).

**Figure 5:**
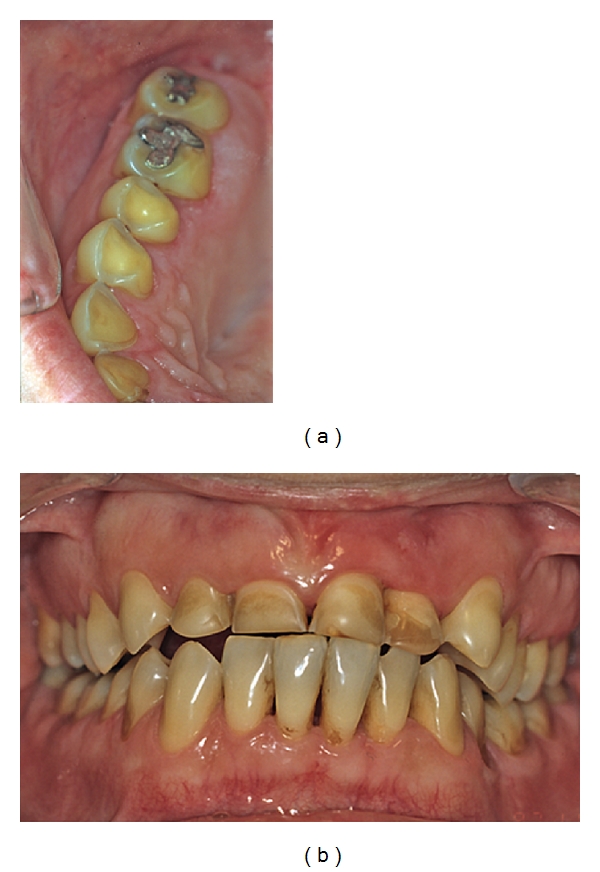
A 40-year-old woman who had suffered from Bulimia Nervosa since she was a teenager. Frequent vomiting followed by intense and meticulous toothbrushing in combination with a high intake of light cola-type soft drinks have resulted in severe erosive tooth wear. A number of “raised” amalgam fillings have developed resulting in an unstable occlusion. At the time that these photographs were taken, she had for a long time been free of her eating disorder but suffers a lot from tooth sensitivity [11].

**Figure 6:**
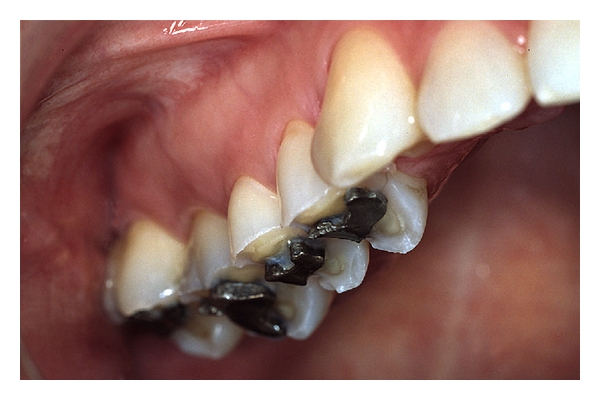
A 40-year-old woman with inoperable hiatus hernia, and despite long-term antireflux medication developed severe damage on her posterior teeth. Note the relatively intact amalgam fillings “raised” above the eroded occlusal surfaces [11].

**Figure 7:**
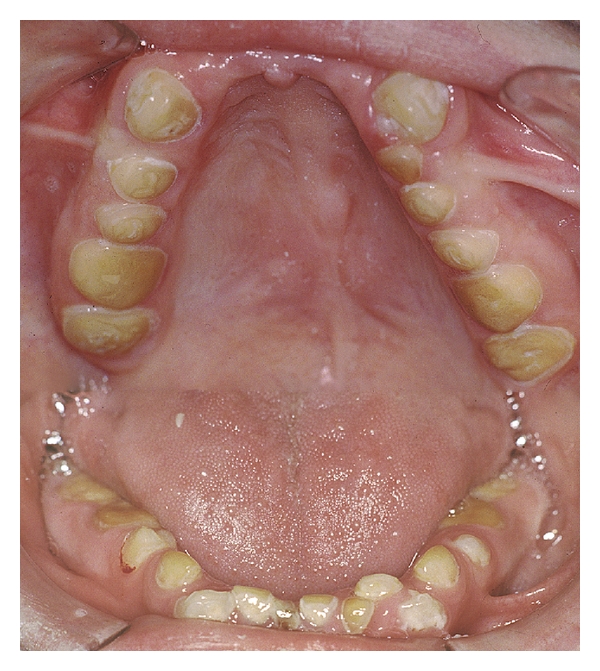
Very severe erosive damage in an intellectually disabled 17-year-old boy with a habit of frequent rumination. In addition to rumination he also suffers from GERD [11].

The “intrinsic” factors include various diseases and habits, which lead to an influx of acidic stomach content into the oral cavity and, so, influencing and/or affecting the teeth. In these cases, for example in patients suffering from eating disorders and gastroesophageal reflux disease (GERD), vomiting and regurgitation, there is an increased risk for erosion [45–49] (Figures 5 and 6). A comprehensive review of GERD appears in this Special Issue [50]. Rumination is a special form of gastric disease which is believed to affect mainly intellectually disabled patients, although its occurrence in the normal population may have been underestimated [51]. The condition involves GERD in combination with voluntary or involuntary regurgitation of swallowed solid food which is then rechewed and reswallowed; the erosive damage might well be severe (Figure 7).

A large number of diseases and syndromes are associated with dental erosion. While the background to this can be that both “extrinsic” and “intrinsic” factors are at play, the net effect may be that acid reaches the tooth surface while there is also present a deterioration in the quantity/quality of saliva, a reduction of oro-motor function, various medications, or mouth breathing. Examples of these, in addition to GERD and eating disorders, are diabetes, high blood pressure, cerebral palsy, salivary gland agenesis, Sjögren's and Down syndromes, and drug abuse, such as alcohol and ecstasy, but not excluding the caffeine-dependence that cola drinks can induce [52–55].

One consequence of the modern lifestyle and the various lifestyle-related diseases of today is that the dentition is more frequently than earlier times, exposed to acidic challenges and the consequent increased risk for dental erosion [56]. This somewhat sudden change in lifestyle, resulting in more acidic challenges for the dentition than earlier, can be compared with the lifestyle change following the 2nd World War that caused a large increase in sugar consumption, which was associated with the subsequent increase in the incidence of dental caries [34]. Although knowledge about dental erosion has improved in recent years, there is an urgent need for further research in order to better and more fundamentally understand its occurrence.

## 3. Clinical Characteristics of Dental Erosion

Dental erosion is defined as “loss of dental hard tissue by a chemical process that does not involve the influence of bacteria” [57]. It occurs as a result of acidic attacks during simultaneous unsaturation of both hydroxyl- and fluor-apatite in saliva, causing loss of dental hard tissue, layer by layer [58]. Early enamel erosion causes no clinical discoloration or softening of the tooth surface and is, therefore, in the clinical situation, difficult to detect both visually and/or by tactile examination. In addition, any patient symptoms, in these early stages, are often absent or very limited. More pronounced changes in macromorphology occur when the erosive damage is more severe. The condition will then be easier to recognize and more likely to present symptoms [2] as well as affecting the oral health-related quality of life of patients [33].

Earlier it was stated that an eroded surface always gave the impression of being a matt surface [59]. It was also stated that dental erosion only could be diagnosed on teeth that had no opposing occlusal contacts [60]. Today it is understood that the surface appearance of an erosive lesion is either blank or matt and that erosion can be diagnosed even if the tooth surfaces have opposing occluding contacts. The erosive lesion can be uneven and produce small concavities. Most often, however, the surface is slightly rounded or flat and sometimes it gives the impression of having “melted” [25] (Figure 3(b)).

**Figure 8:**
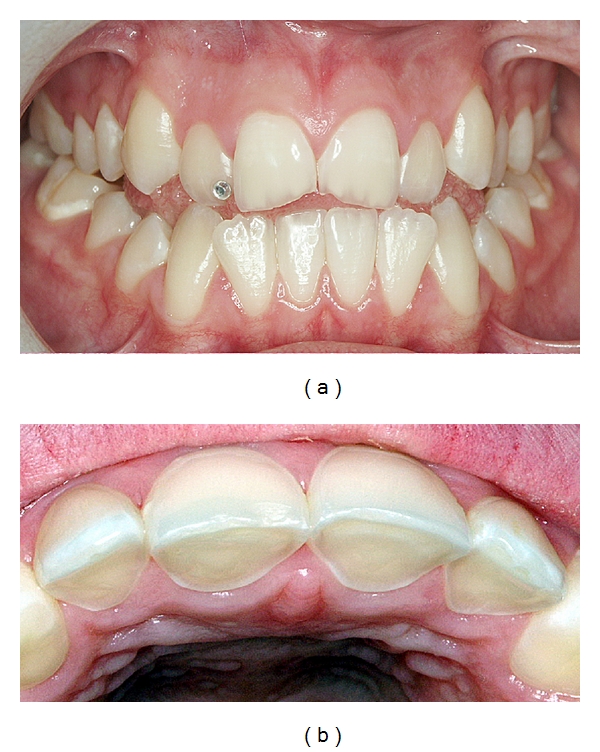
A 13-year-old girl who has a high intake of soft drinks. (a) Buccal erosion and crown shortening of the maxillary front teeth. Note the typical “inverted V-sign” often seen in cases of soft-drink-induced dental erosion. Mandibular incisors are relatively intact. (b) Severe erosive damage, with shoulder formation on the palatal surfaces of maxillary anterior teeth. Published with permission from the Journal of the Swedish Dental Association (Tandläkartidningen) [12].

**Figure 9:**
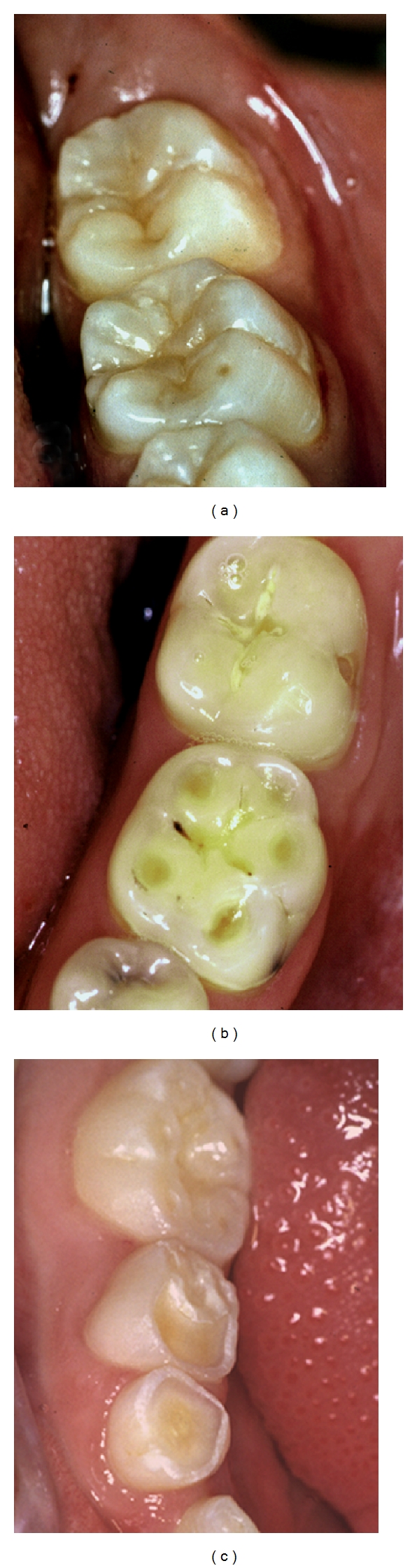
Examples of “cupping” of different severities in 3 individuals. (a) Cuppings of lesser extent on 36 mesiobuccal cusp in a 20-year-old man who has high intake of cola drinks. (b) Cuppings on 36 in a 22-year-old man with congenital agenesis of salivary glands. (c) Fused cuppings on teeth nos. 84 and 85 in a 5-year-old boy. Published with permission from the Journal of the Swedish Dental Association (Tandläkartidningen) [12].

**Figure 10:**
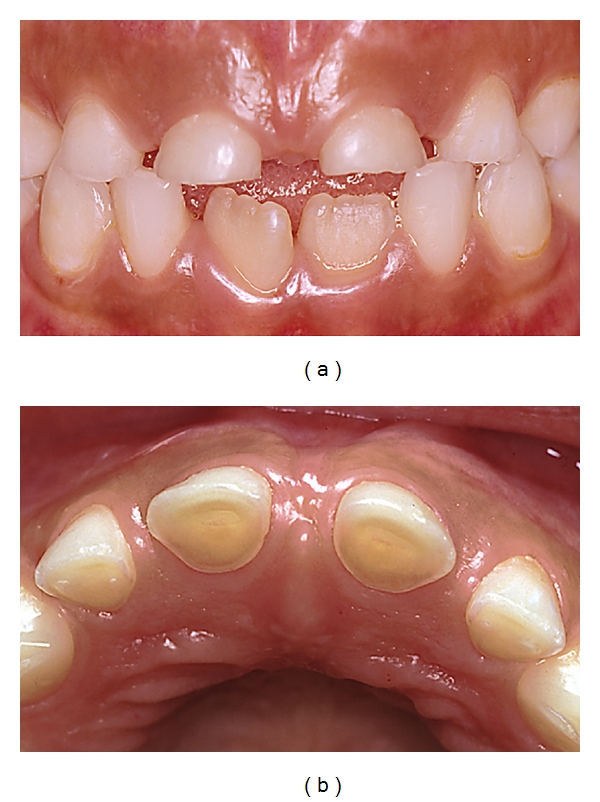
Erosion in the primary dentition in a 6-year-old girl who has high intake of juice, fruit drink, cola, and fruit. (a) Shortening of the crown height on teeth nos. 51–61 as a consequence of dental erosion. (b) Note that the pulp is visible through the remaining tooth substance of teeth nos. 51–61 palatally. Published with permission from the Swedish Dental Journal [2].

In today's populations, dental erosion occurs on all tooth surfaces but is common palatally on maxillary anterior teeth and on occlusal surfaces of lower first molars [25, 61]. Proximal erosive lesions are difficult to diagnose but are probably rare, whereas a cervical shoulder formation (Figures 4(a) and 8(b)), as well as the reversed V-sign incisally on maxillary central incisors are more common (Figure 8(a)). “Cupping” is a concavity in the enamel, usually on a cusp tip, with or without dentinal involvement, is a common sign of dental erosion, and in posterior teeth they are usually located on first molars especially in the lower jaw (Figure 9). Cuppings are strongly correlated with dental erosion and should be carefully looked for as it has been stated that they can be regarded as an indicator of the onset of erosion [62]. In advanced cases of erosion, the pulp can be visible through the remaining tooth substance. This is especially the case in the maxillary central incisors in the primary dentition but can also be seen in the permanent dentition (Figures 3 and 10).

On the basis of archeological data, populations from the past experienced endodontic complications that were mostly related to tooth wear, whereas today they are most often related to dental caries [63]. While progression of the wear observed in skull materials was considered as “linear,” indeed even allowing age determination [64], the wear in contemporary populations may progress as a combination of “linear” deterioration, but also superimposed “bursts,” possibly coinciding with the presence of certain lifestyle factors or lifetime events [65]. Since episodic wear does not preclude the occurrence of a more slowly progressing “background” wear of the occlusal surfaces, it adds a further dimension to the phenomenon of wear [1], and, most importantly, to its management. It will be evident that the loss of tooth substance brought about by dental erosion may at some stage present dissatisfaction for patients; it is to be hoped that at an early enough stage the attending dentist will have identified the matter, informed the patient and implemented an initial preventive strategy. However, actual complaints from the patient are more likely in the later stages, resulting in aesthetic, orthodontic, and functional complications and/or be associated with sensitivity and pain [66], most of which will require the consideration of restorative and other interventions.

## 4. Other Types of Tooth Wear and Their Relation to Dental Erosion

Tooth wear has a multifactorial etiology and is usually a result of more than a single mechanism [67]. In addition to erosion, other types of wear can occur in parallel. This includes, for example, attrition (tooth wear caused by contact between occlusal/incisal surfaces) and abrasion (tooth wear caused by a foreign body such as a toothbrush, or biting on a hairpin or pencil).

It is well recognized that enamel recently softened by acid will/may/can wear easier by concurrent mechanical impact, compared to enamel that was not so softened [68]. Today, there is evidence that a key element in a severely worn dentition is erosion, and that attrition and abrasion are of lesser importance [1, 69–71].

There is no strong support for the (previously) common belief that bruxism is the main cause of tooth wear. It has been shown that in individuals with tooth wear and simultaneous bruxism, erosion, and not attrition (bruxism) is the dominant etiological factor related to the loss of tooth substance [72]. Whether bruxism is a causative factor in tooth wear is still not fully understood, but it is fair to state that it's role has probably been overestimated [73]. If strict diagnostic assessment of bruxism is carried out (e.g., polysomnography), no clear conclusions can be drawn about the role of either bruxism in tooth wear or in relation to failing restorations [73, 74]. In addition, it is feasible, even likely, that the tongue can influence the loss of tooth surface by abrasive action on teeth, following their “softening” by an acid attack. Whether the tongue might also act as a reservoir for acid after an acidic challenge has been discussed. This may not be the case after intake of an acidic drink since the pH on the tongue surface recovers very quickly after drinking [66].

### 4.1. Noncarious Cervical Lesions

Noncarious cervical lesions (NCCLs) are by tradition often considered equivalent to toothbrush abrasion. However, research has shown that the cause of these injuries cannot be blamed on intense or improper brushing techniques alone, as they may occur subgingivally, in individuals who seldom brush their teeth, in archeologically recovered material (clearly before the toothbrush era), and even in animals [25, 75–80].

Definitions of the various forms of NCCL are often imprecise which may be one explanation for the wide range of prevalences reported. Three types can be easily observed and/or be discernible, namely, a shallow cervical lesion, a grooved cervical lesion, and a wedge-shaped cervical lesion [56]. The most common NCCL is the shallow cervical erosion. NCCLs appear to develop with age from a shallow lesion into the other types mentioned, with wedge-shaped lesions being most prevalent in older adults. NCCLs are common on the facial and buccal surfaces of the maxillary anterior and premolar teeth and the mandibular premolar teeth. [56].

Significant correlations have been found between NCCLs and the presence of occlusal erosive lesions as well as occlusal attrition [2, 81]. Wedge-shaped NCCLs have been called abfractions, assumed to be caused by heavy stress on the teeth (namely, due to heavy chewing or bruxism), or in combination with an acidic challenge which will result in strain microfractures along the buccal cemento-enamel junction, making the area more prone to substance loss when stressed [76, 82, 83]. On the other hand, wedge-shaped cervical lesions have also been identified on teeth without occlusal contacts. The theory has, not surprisingly, received criticism due to lack of robustness of the evidence [84].

A review concluded that toothbrushing, with or without toothpaste, only minimally contributes to the development of wear of enamel, whereas toothbrushing in combination with an acidic diet may be linked to dentin wear and hypersensitivity [85]. It is likely that NCCLs not only have a multifactorial aetiology, including many factors besides toothbrushing [86], but also that toothbrushing in the presence of acid may contribute to a more rapid development of NCCLs [85, 87]. As there is near consensus today that the most important etiological factor for NCCLs is erosion, preventive measures to reduce acidic challenges on the teeth is essential in managing patients with NCCLs.

## 5. Diagnosis and Grading of Dental Erosion in the Clinic

For the purposes of appropriate clinical decision making, it is necessary to quantify the severity of erosion at a certain point of time, as well as the progression of erosion during a specific time interval. Different techniques are available, ranging from sophisticated optical or laser scanning methods to relatively simple ordinal scales [88]. The latter scales are mostly designed for epidemiological studies, but can be appropriately adapted for clinical use. Examples of such scales are shown in Tables 2 [25] and 3 [1]. More recently, digital scanning systems, now on the market, may offer great opportunities as scanning can be performed intraorally, on study casts and impressions. Software is now available which allows superimposition of scanned images from different occasions in order to assess the amount and rate of tooth substance loss.

Different scales have been used in different systems for clinical examination and diagnosis of dental erosion. The systems have different approaches to score surface loss in enamel and dentin. There is no consensus whether dentin exposure is an adequate measure of the severity of erosive damage, given that the existence of dentinal exposure is in itself very difficult to diagnose clinically [89].

Approaches to diagnosis and recording are/have been based upon cervical, bucco-palatal, or inciso-occlusal surfaces, and full mouth/partial mouth or combinations thereof. In addition, selection criteria, sampling technique, and age composition have varied. Data obtained from different studies are, therefore, often difficult to compare, which leaves the epidemiological basis for the occurrence of dental erosion uncertain [90]. Both full mouth recording, involving grading of all teeth, and partial recording, involving only specific marker teeth, may be used. Generally, full mouth recording is more time consuming for both dentist and patient and is more feasible for research than for routine clinical use. The marker teeth to be used for clinical recording should be ones that are commonly affected by erosion as well as having easily detectable clinical features (when erosion is present). In this regard, a simplified erosion partial recording system (SEPRS) has been developed, namely, using maxillary central incisors palatally and cuppings on lower first molars in the permanent dentition (total 4 surfaces) and maxillary central incisors palatally and cuppings on all four molars in the primary dentition (total 6 surfaces) (scoring by using Tables 2 and 3). By using this system, specificity and sensitivity close to 100% were obtained in relation to scoring of all maxillary canines/incisors and first permanent/all primary molars [23].

## 6. Prevalence of Dental Erosion

Cross-sectional population studies of dental erosion have reported a varying prevalence (Table 1). The interpretation of early studies was that the presence of erosion was increasing among children and the youth, but this was with some reservation as comparative/longitudinal studies did not exist. That the most severe damage found was on the palatal surfaces of maxillary anterior teeth caused some confusion since the focus had previously been on occlusal and incisal wear. Today, it has been confirmed in many countries, that dental erosion, particularly palatal damage of the upper front teeth, are common among children and young people.

Recent longitudinal studies in children and adolescents are of special value given the limitations of noncomparability of earlier studies. A longitudinal study from Germany showed an increase in erosive damage in children between 1977–87 and 1990–99. The number of lesions nearly doubled during this period of time; erosion into dentin on at least one primary tooth increased from 18 to 32%, and on the first mandibular molars from 4 to 9% [8]. Similar findings have been reported in British adolescents [91]. In the UK, 27% of the 12-year-olds had developed new or more advanced erosive damage at age 14. Lesions into the dentin were noted in 5% of 12-year-old children, which by the age of 14 had increased to 13%. The corresponding figures for erosion confined to the enamel were 56 and 64% [9]. In a recent study from the Netherlands that followed children from the age of 12 to the age of 15, the incidence of new dental erosion decreased during this 3-year period while the prevalence of deep enamel/dentin erosion increased from 2% to 24% in children who already had erosion at the age of 12 years [10].

### 6.1. Relationship between Cuppings, NCCLs, and Dental Erosion

The presence of cuppings on first molar teeth is widely accepted as a clinical sign of erosion. In a sample of Saudi young men, cuppings on the first molars were found in 49% of individuals [25]. In high and low erosion groups from the same sample [25] the prevalence of cuppings was 64% and 41%, respectively [42]. German children with erosion showed 87% cuppings at age 11 and 94 percent at the age of 16 [8]. Australian studies have shown that cuppings are both more frequent and larger in size in erosion patients younger than 27 years compared to older erosion patients. This has been interpreted as a result of a lifestyle change that led to an increased consumption of acidic drinks, a choice which was seen as far more pervasive among youngsters [62]. Prevalences of cuppings in 5-6-year-old, 13-14-year-old and 18-19-year-old Swedes were 72%, 46%, and 66%, respectively, and had a significant correlation with the presence of erosion on anterior maxillary teeth [23].

NCCLs were noted in 25% in an unselected material of young men, while in patients groups with high and low erosion the prevalence was 58% and 10%, respectively [42]. Among Swedish children at the age of 5-6 years, NCCLs were found in 44% of the individuals, among 13-14 years, in 87%, and among 18-19 years, in 98% [23]. In the latter study, a significant correlation between mean erosion scores on maxillary anterior permanent teeth and number of buccal NCCLs was found for the groups of 13-14 years and 18-19 years but not for primary teeth (group 5-6 years). Others have made similar findings [81]. A quarter of the subjects in one study showed wedge-shaped lesions, abfractions, and 5% of all teeth exhibited such lesions [86] and if other more indistinct types of NCCLs were included, a prevalence of 62% was reported of the subjects investigated [87].

## 7. The Individual's Defense against Erosion

Studies of both primary and permanent teeth have shown that tooth surface hardness plays a role in the development of erosive damage. Although primary teeth are softer than permanent teeth, the erosive process progresses at the same rate on both types. Children have larger variations and slower salivary sugar clearances and also lower salivary flow rates than adults. In addition, primary teeth are “smaller” than permanent teeth. Considering hard tissue morphology, quality of saliva, and other salivary conditions, the erosion of deciduous teeth is therefore likely to manifest more quickly than it does on permanent teeth [92–96].

Saliva is one of the most important defense mechanisms for dental erosion. Oral clearance of an acidic product varies individually with the salivary secretion rate but also by the individual's ability to swallow. It has been demonstrated that a dry-mouth individual runs a higher risk of erosion than an individual with normal salivary secretion rate [97], and that children with erosion, despite having low caries activity, have saliva with properties similar to saliva of children with high caries activity [98]. It has also been suggested that salivary buffering capacity is of greater importance in cases of erosion compared to that of dental caries [99]. In this respect, it should be noted that children normally have a lower salivary secretion rate than adults [100] and also a lower capacity of swallowing.

The pellicle that saliva forms on teeth varies in thickness not only between individuals but also between different locations in the mouth. Studies have shown that the salivary pellicle, depending on its thickness, offers some protection for acid erosion on enamel [101, 102]. However, on a newly eroded surface a rapid buildup of new pellicle will adapt strongly to the eroded surface, thus hindering remineralization. The capacity of a pellicle to protect against erosion was shown to be limited in the face of a weak acidic challenge on enamel, and nonexistent for such a challenge on dentin [103]. It will be clear that the various factors influencing pellicle and plaque formation can be decisive for where erosive damage will occur, and for its severity. It has been suggested that different protein interactions may be of importance in this regard [104], one such factor being salivary concentration of urea [66, 105].

The importance of method of consumption has been investigated regarding drinking. The method of drinking, namely, how an acidic beverage is drunk, is of great importance for the outcome of the erosive attack. A “retaining” drinking technique, that is, keeping the drink in the mouth before it is swallowed increases the risk of erosion as the contact time between the tooth and drink is extended. It has been shown by clinical research that patients with high erosion more frequently use a “retaining” drinking technique than patients with a low degree of dental erosion. Method of drinking is assumed to depend on many different factors, such as perceived taste, quantity of carbonic acid and the ability to swallow [66, 106] and maybe even to behavioral aspects.

Oral hygiene habits are correlated with erosion, and especially so if they are carried out in conjunction with an ongoing acidic attack on the surfaces of the teeth [107]. It has been suggested that the acid-induced, softened tooth surface needs about an hour in the presence of saliva to remineralize, and so be better able to resist abrasion from toothbrushing [108]. It has been reported that patients with erosion often have good gingival conditions and a small amount of plaque [66]; it is also known that a more methodical, rigorous oral hygiene technique is associated with erosion to a greater extent than does a more sporadic and less systematic method [42]. That being so, this is not meant to imply that the proven benefits of good oral hygiene practice should be compromised “for the purpose of avoiding erosive wear.”

## 8. Lifestyle and Behavioral Factors and Dental Erosion

It is well known that both oral health and general health are affected by lifestyle and behavioral factors [109–111]. Lifestyle change over time and often reflects societal factors. These factors commonly include food choices and drinking habits, level of physical activity, stress-related disorders, and/or abuse of substances, amongst others.

A significant change in today's lifestyle, as mentioned earlier, is the sharply increasing consumption of acidic beverages mainly in groups of children and young people [41, 112, 113]. Another example is that people choose a new “healthy lifestyle,” the net, unplanned outcome being that they have a diet with an increased content of acidic products. Examples of this are vegetarians and those who diet or fast in order to lose weight [114–116]. The desire to keep fit can be tied to the need to drink plentifully when training in the gym, on the track, or at home; the risk here is of taking a sour drink, and often under conditions of a deteriorated salivary condition [117, 118].

A more “unhealthy” lifestyle can similarly have negative implications for the risk of erosion. Stressed, perhaps over-worked, take a quick lunch “on-the-run,” control the heartburn that they likely to have with medication that may relieve a gastrointestinal problem, but can also lead to reduced salivary secretion [119]. Similarly, drug abusers [120–122], those, such as the young IT freak who stays awake through the night with the help of a caffeine-containing cola drink, and others living relatively “unhealthy” lives, are at risk for erosion. Prevalence of dental erosion appears not to follow a clear socioeconomic pattern [16, 123, 124] or to demonstrate direct sex differences [16, 24, 125, 126], although it does vary between different age groups. In China, 3–5 year-old children generally show a low prevalence of erosive tooth surface loss. The children with erosion had parents with higher education who also, to a greater extent than others, embraced the Western way of life and let their children have frequent intakes of fruit drink in the baby bottle just before bedtime [21].

The complex interactions among social, behavioral, and circumstantial factors that contribute to the development of dental erosion are very important considerations in furthering our understanding of, and managing, the condition. Although this is a complicated task, the growing challenge the condition poses requires that every effort be made in this research direction.

## 9. Clinical Aspects

Attempts have been made to develop grading systems of the severity of wear that would have applicability in the clinical setting. A recent development is a screening and monitoring system (BEWE) that is based on treatment need categories [127]. The proponents regard the system as an important tool that has the possibility to become an integral part of the regular oral and dental clinical examination that patients undergo at routine recalls; the benefits, they imply, outweigh the oversimplification of a complex and varied condition. Thus, there has been strong criticism leveled against the BEWE system due to several shortcomings: for example, not considering pain, sensitivity or poor aesthetics, which are crucial factors in the clinical decision making determining the need for intervention, or not [128]. The assessment of the need for treatment for erosive damage must always be made on an individual basis taken into account a multitude of factors. This means that the same degree of damage can be in need of treatment in one patient but not in another. Naturally, such complex decisions cannot be made from a scoring system only. A patient diagnosed with erosion should be followed up with individualized recall periods, and an assessment of the possible progression from the different investigations should be made. If necessary, a medical consultation and/or complementary medical examination should be carried out.

### 9.1. Preventive Strategies as the First Line in Management

Prevention will frequently involve a need for lifestyle changes, not only for the individual but also for the whole family. To effectively eliminate or reduce the acidic effect on teeth is clearly of far greater value than, for example, recommending treatment with different fluoride products. These have a more limited clinical effect in dental erosion [129–136], even if their positive effect in the prevention of dental caries is well established. The use of neutralizing products such as antacid drugs has been shown to increase intraoral pH after an acidic challenge [137, 138], while rinsing with bicarbonate decreased tooth surface loss after artificially induced erosion, it did not alter surface microhardness [139]. A large variety of dental care products for prevention of dental erosion is on the market but there is no formula or product available today that provides adequate protection against erosion [140]. A product that may be promising, however, may be one containing casein phosphopeptide-amorphous calcium phosphate (CPP-ACP) [141].

The erosive potential of food and drinks is a measure of its capacity to demineralize tooth substance. In general, erosive tooth wear on enamel takes place if the pH is below 5.5. However, studies have shown that modification of this critical pH is possible by addition of, for example, calcium or phosphate to drinks [142, 143]. It does not appear that this method of prevention is commonly applied today.

The young child with eroded primary teeth is a challenge. It may, however, give an opportunity for preventing erosion in the permanent dentition. Advice and information about dental erosion at the right time can, in many patients, fully or partly, prevent further damage, while in other patients it can be less successful. However, it has been shown that even in cases of severe erosion, for example in connection with eating disorders, information and prophylaxis can reduce the risk for development of erosive damage [144]. To promote a healthier behavior among adolescents, their choice of lifestyle needs further investigation and collaboration between different types of healthcare workers.

### 9.2. Restorative Treatment

It would not be an overstatement to say that all clinical decision-making related to interventional procedures come down to a careful weighing of the benefits and the risks of the treatment options being considered. The restoration of teeth with tooth surface loss is no exception, and it is certainly not necessary to restore all cases of tooth surface loss. Furthermore, there are no clear guidelines for appropriate restorative treatment of erosive damage, and there is a striking lack of evidence regarding the long-term outcomes of any particular treatment methods and material. All this calls for caution in clinical decision-making [73]. In the young dentition, composite restorations constitute the mainstay of restorative interventions, while expensive conventional fixed and removable prosthodontics was, and still remains, at the center of rehabilitation of the extensively worn adult dentition—when treatment is indicated. Such treatment is also complex and generally highly invasive, adding to the dilemma of having to further remove tooth substance for retentive needs when faced with already an erosively-reduced tooth. To reemphasize the previous section, prevention is the golden rule for success.

**Figure 11:**
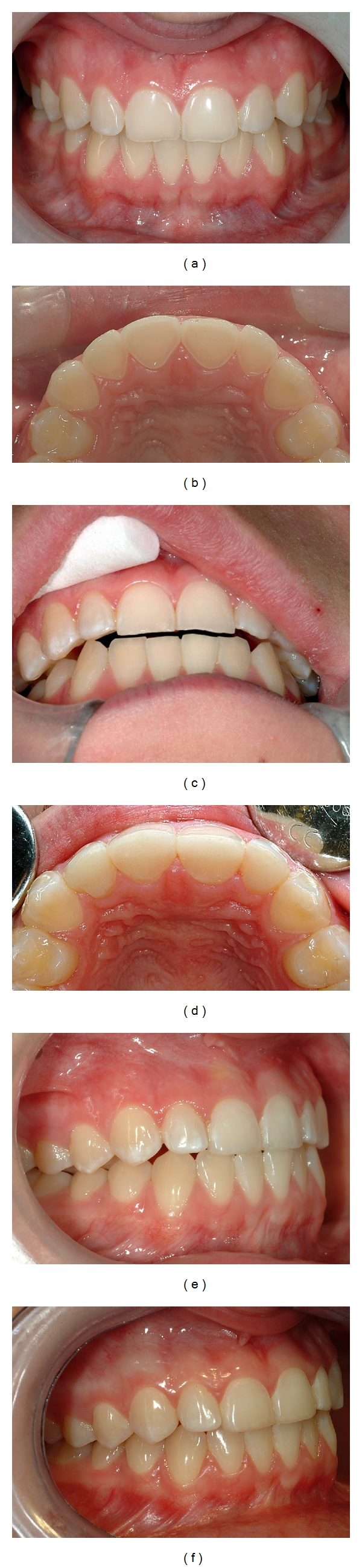
(a) A 15-year-old girl with dental erosion confined mainly to maxillary anterior teeth caused by excessive soft drink consumption. (b) Substantial loss of toots substance palatally on teeth nos. 12–22 with shoulder formation. (c) Vertical dimension established by composite restoration on 12 allowing adequate space for restorative material. (d) Coverage with composite on teeth nos. 13–23. (e) Just after placement of composite. Note the nonoccluding posterior teeth. (f) After a short period of time, reestablishment of the posterior occlusion utilizing the Dahl principle [11].

Luckily, in many cases of wear and especially in the young patient, the focus of restoration will be concentrated to the anterior segment of maxillary teeth, usually for aesthetic reasons, but not exclusively so. The problem of restoring worn anterior teeth when little available interocclusal space exists is apparent. In this regard, a less radical alternative to complete occlusal reconstruction, based on the principles of combined forced intrusion of anterior teeth and supra-eruption of posterior teeth was first described by Dahl et al. [145]. The method has consistently been shown to be reliable in clinical studies [146–149]. Most children and adolescents deemed to require restorative treatment can be treated with a Dahl approach, or modifications thereof. Several such modifications have been described following the original report, including placement of single- or multiple-bonded restorations at increased vertical dimension of occlusion (VDO) in anticipation of rapid reestablishment of full intercuspation being attained by the nonoccluding teeth [148]. Thus it can be seen that, besides the traditional “subtractive” approach of conventional rehabilitative methods, a shift towards greater conservatism through an “additive” approach is underway in the form of direct and indirect resin composite restorations [150–152], as well as other materials. The result can be both esthetically and functionally very satisfactory (Figure 11). In older patients the same approach may well be used. In one study, a shorter life-span with both direct and indirect resin composite was reported in patients with erosion compared to the control group [153]. In other clinical reports, promising results were shown [154, 155].

**Figure 12:**
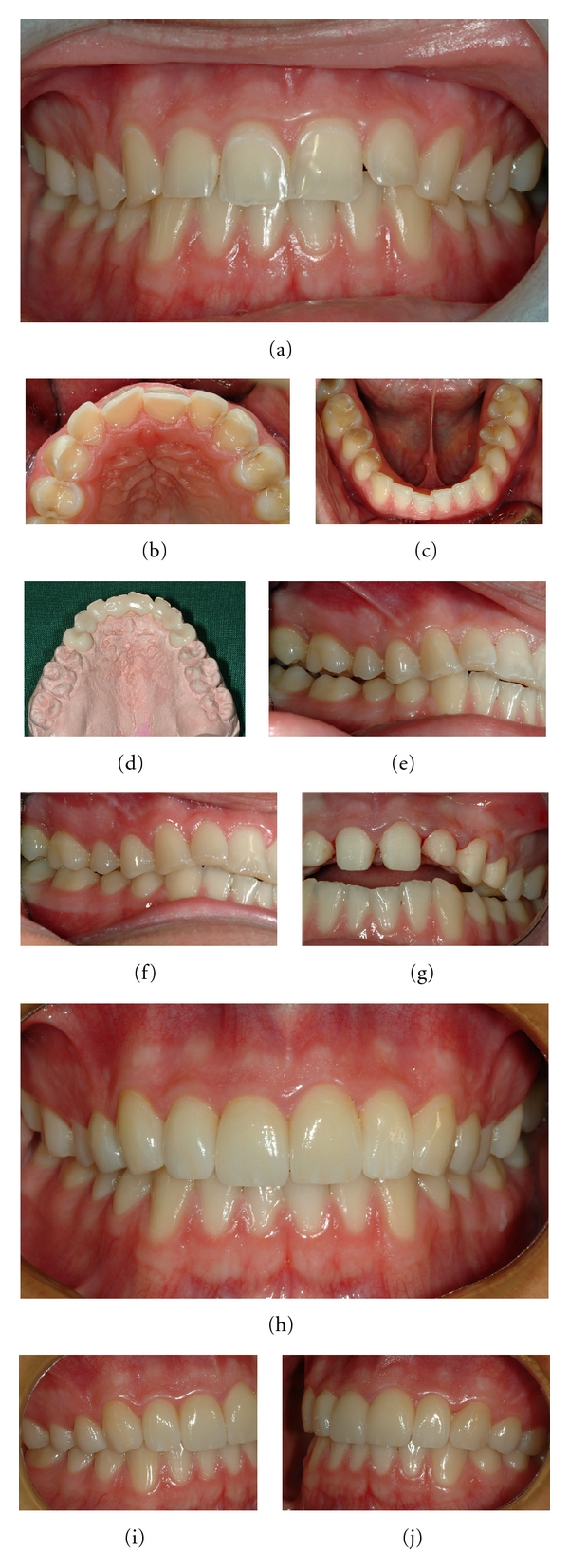
A 19-year-old man with extensive tooth wear affecting maxillary anterior teeth caused by excessive soft drink intake, drunk by the “retaining” drinking technique (a–c). Note the pronounced wear on palatal and buccal surfaces with shoulder formations (b). Patient is provided with palatal acrylic onlays *ad modum* Dahl (cemented with resin cement) producing posterior disclusion (d, e). After 4 months the posterior occlusal relationship has normalized (f) and after preparation there is enough space for the restorations (g). Full ceramic Empress crowns cemented on teeth nos. 14–24 (h–j) [13, 14].

With modern ceramics, using an adhesive technique, similarly good results can be achieved (Figure 12). It seems possible that the all-too-frequent failures seen after traditional reconstructive efforts may be more controllable through a staged, reversible reconstructive approach that relies to a large extent on adhesive technology, for example, bonded composite restorations [152, 156]. Given the possibilities that such innovative approaches offer, it must be acknowledged that clinicians have provided, and continue to provide, rehabilitative strategies for managing their patients' worn dentitions that range traditionally from extensive prosthodontics to an increasing reliance on adhesive techniques. Since all restorations have a finite lifespan, it is necessary for the clinician to know that early diagnosis and preventive strategies should always be used in patients at risk so as to avoid the development of severe wear and the need for complicated, extensive rehabilitation.

## 10. Conclusions

Although it may be difficult to identify a single cause of tooth wear that may be seen clinically, dental erosion is increasingly and more consistently recognized as an essential feature of the wear seen.

The interest in dental erosion has been steadily increasing since the mid-1990s. Dental erosion has a multifactorial background, with individual and lifestyle factors having great significance. Dental erosion is commonly seen in children and young people. The location of erosive lesions is typically on the palatal surfaces of maxillary anterior teeth and the occlusal surfaces of mandibular first molars. Erosion appears also to be a significant factor in the development of noncarious cervical lesions.

It is important for the oral healthcare team to recognize the early signs and symptoms of dental erosion and to understand its pathogenesis. This is key to understanding what the focus of the management approach needs to be.

Preventive strategies are the essential first line in management, and will include lifestyle changes. To promote healthier behaviours in adolescents, their choices of lifestyle will often need further investigation and collaboration between different types of healthcare workers.

When restoration of erosive damage is indicated, it should preferably, and if clinically and technically possible, be based on principles of reversibility. Thus the technique used should be “additive” rather than “subtractive.”

Notwithstanding the greater knowledge base that exists compared to about 15 years ago, there is still a need for further research and a better understanding of dental erosion, be it of the process itself and its causation, or the most appropriate ways to deal with its consequences.

## Figures and Tables

**Table 1:** Prevalence of dental erosion, according to studies from different countries. Prevalences, as listed, refer to erosion that reaches to the dentin or deeper.

Country	Age (yr)	No. of individuals	Prevalence (%)	Author(s) and publication yr
Children				
United Kingdom	4-5	178	30	Millward et al. 1994 [15]
United Kingdom	5	>1000	24	Downer 1995 [16]
United Kingdom	1.5–4.5	1658	8	Moynihan and Holt 1996 [17]
Saudi Arabia	5-6	354	34	Al-Majed et al. 2002 [18]
Ireland	5	202	21	Harding et al. 2003 [19]
India	5-6	100	30	Deshpande and Hugar 2004 [20]
China	3–5	1949	1	Luo et al. 2005 [21]
Germany	2–7	463	13	Wiegand et al. 2006 [22]
Sweden	5-6	135	13	Hasselkvist et al. 2010 [23]
Adolescents				
United Kingdom	14	1035	30	Milosevic et al. 1994 [24]
United Kingdom	15	>1000	2	Downer 1995 [16]
Saudi Arabia	20	95	16	Johansson et al. 1996 [25]
Cuba	12	1010	17	Kunzel et al. 2000 [26]
Saudi Arabia	12–14	862	26	Al-Majed et al. 2002 [18]
Iceland	15	278	6	Arnadóttir et al. 2003 [27]
United Kingdom	14	1308	13	Dugmore and Rock 2003 [9]
United Kingdom	14	2351	53	Bardsley et al. 2004 [28]
Turkey	11	153	28	Caglar et al. 2005 [29]
Brazil	12	389	2	Correr et al. 2009 [30]
Netherlands	15	622	24	El Aidi et al. 2010 [10]
Iceland	12	757	1	Arnadottir et al. 2010 [31]
Iceland	15	750	6	Arnadottir et al. 2010 [31]
Sweden	13-14	227	12	Hasselkvist et al. 2010 [23]
Sweden	18-19	247	22	Hasselkvist et al. 2010 [23]
Adults				
Switzerland	26–30	197	11	Lussi et al. 1991 [32]
Switzerland	46–50	194	19	Lussi et al. 1991[32]
United Kingdom	22	1010	77	Daly et al. 2011 [33]

**Table 2:** Ordinal scale used for grading severity of dental erosion on buccal and lingual surfaces of maxillary anterior teeth [25].

Grade	Criteria
0	No visible changes, developmental structures remain, macromorphology intact.
1	Smoothened enamel, developmental structures have totally or partially vanished. Enamel surface is shiny, matt, irregular, “melted,” rounded or flat, and macromorphology generally intact.
2	Enamel surface as described in grade 1. Macromorphology clearly changed, facetting or concavity formation within the enamel, no dentinal exposure.
3	Enamel surface as described in grades 1 and 2. Macromorphology greatly changed (close to dentinal exposure of large surfaces) or dentin surface exposed by ≤1/3.
4	Enamel surface as described in grades 1, 2 and 3. Dentin surface exposed by >1/3 or pulp visible through the dentin.

Note: approximal erosion, presence of “shoulder” and “cuppings” should be recorded.

**Table 3:** Ordinal scale used for grading cuppings on occlusal surfaces of first permanent molars and primary molars [23].

Grade	Criteria
0	No cupping/intact cusp tip
1	Rounded cusp tip
2	Cupping ≤ 1 mm
3	Cupping > 1 mm
4	Fused cuppings: at least two cuppings are fused together on the same tooth
